# Changes to virus taxonomy, the international code of virus classification and nomenclature, and the ICTV statutes ratified by the International Committee on Taxonomy of Viruses (2025)

**DOI:** 10.1007/s00705-025-06485-1

**Published:** 2025-12-18

**Authors:** Peter Simmonds, Evelien M. Adriaenssens, Elliot J. Lefkowitz, Hanna M. Oksanen, Francisco Murilo Zerbini, Poliane Alfenas-Zerbini, Frank O. Aylward, Donald M. Dempsey, Juliana Freitas-Astúa, R. Curtis Hendrickson, Holly R. Hughes, Mart Krupovic, Jens H. Kuhn, Małgorzata Łobocka, Richard Mayne, Arcady R. Mushegian, Judit J. Penzes, Alejandro Reyes Muñoz, David L. Robertson, Simon Roux, Luisa Rubino, Sead Sabanadzovic, Donald B. Smith, Nobuhiro Suzuki, Dann Turner, Koenraad Van Doorslaer, Arvind Varsani

**Affiliations:** 1 Virology Institute of Biomedicine, University of Turku, Kiinamyllynkatu 10, Turku, FI-20520 Finland; 2 Quadram Institute Bioscience, Norwich Research Park, Norwich, NR4 7UQ UK; 3 Department of Microbiology, University of Alabama at Birmingham, ALGEN 550, 701 19th St South, Birmingham, AL 35294 USA; 4 Molecular and Integrative Biosciences Research Programme, Faculty of Biological and Environmental Sciences, University of Helsinki, Viikinkaari 9, Helsinki, 00014 Finland; 5 Departamento de Fitopatologia/BIOAGRO, Universidade Federal de Viçosa, Viçosa, MG 36570-900 Brazil; 6 Departamento de Microbiologia, Universidade Federal de Viçosa, Viçosa, MG 36570-900 Brazil; 7 Department of Biological Sciences, Virginia Tech, Blacksburg, VA USA; 8 Embrapa Cassava and Fruits, Cruz das Almas, BA 44380-000 Brazil; 9 Centers for Disease Control and Prevention, Fort Collins, Colorado USA; 10 Institut Pasteur, Université Paris Cité, CNRS UMR6047, Cell Biology and Virology of Archaea Unit, 25 rue du Dr Roux, Paris, 75015 France; 11 Integrated Research Facility at Fort Detrick, National Institute of Allergy and Infectious Diseases, National Institutes of Health, B-8200 Research Plaza, Fort Detrick, Frederick, MD 21702 USA; 12 Institute of Biochemistry and Biophysics of the Polish Academy of Sciences, 02-106 Warsaw, Poland; 13 Nuffield Department of Medicine, University of Oxford, Peter Medawar Building, South Parks Road, OX1 3SY Oxford, UK; 14 Bethesda, MD 20814 USA; 15 Department of Entomology, Texas A&M University, College Station, TX 77843 − 2475 USA; 16 Departamento de Ciencias Biológicas, Universidad de los Andes, Bogotá, Colombia; 17 MRC-University of Glasgow Centre for Virus Research, Sir Michael Stoker Building, 464 Bearsden Road, Glasgow, G61 1QH UK; 18 DOE Joint Genome Institute, Lawrence Berkeley National Laboratory, Berkeley, California USA; 19 Consiglio Nazionale delle Ricerche, Istituto per la Protezione Sostenibile delle Piante, Sede Secondaria di Bari, Via Amendola 165/A, Bari, 70126 Italy; 20 Department of Agricultural Science and Plant Protection, Mississippi State University, Mississippi State, MS 39762 USA; 21 Institute for Genomics, Biocomputing and Biotechnology, Mississippi State University, Mississippi State, MS 39762 USA; 22 Institute of Plant Science and Resources, School of Applied Science, College of Health, Science and Society, Okayama University, Kurashiki, Okayama 710 − 0046 Japan; 23 University of the West of England, Bristol, BS16 1QY UK; 24 Department of Immunobiology, School of Animal and Comparative Biomedical Sciences, BIO5 Institute, University of Arizona Cancer Center, Tucson, AZ 85721 USA; 25 The Biodesign Center for Fundamental and Applied Microbiomics, School of Life Sciences, Center for Evolution and Medicine, Arizona State University, Tempe, AZ 85287 − 4701 USA

**Keywords:** Taxonomy proposal, Ratification vote, Realm, Phylum

## Abstract

The 56th meeting of the Executive Committee (EC) of the International Committee on Taxonomy of Viruses (ICTV) was held in Bari, Italy, in July/August, 2024, and 115 submitted taxonomy proposals were reviewed. A total of 112 were subsequently ratified by the ICTV membership. An additional 9 error correction proposals were also approved in August 2025. This article lists the taxonomy proposals that have now been incorporated into release 40 version v2 of the Master Species List (https://ictv.global/msl), the Virus Metadata Resource (https://ictv.global/vmr), and associated ICTV databases. In addition to the assignments of 1,563 new virus species, 243genera, 55 families, 11 orders, and 8 classes, there were substantial additions to higher taxonomic ranks. These include the creation of a new realm (*Singelaviria*), which is based on the recognition of a separate evolutionary origin for the hallmark capsid genes of members of the kingdom *Helvetiavirae.* These express capsid proteins forming a single jelly-roll fold that is structurally and evolutionarily distinct from those of members of the family *Bamfordvirae*, assigned to the realm *Varidnaviria*. Furthermore, the realm *Varidnaviria* underwent a major reorganization, including the addition of a new kingdom, *Abadenavirae*. Another notable change was the classification of the vertebrate-infecting single-stranded DNA anellovirids into a new phylum *Commensaviricota* (kingdom *Shotokuvirae*, realm *Monodnaviria*). Archaeal viruses infecting the hyperthermophilic *Archaeoglobi* were assigned to a new phylum *Calorviricota*, in the kingdom *Trapavirae* (realm *Monodnaviria*), whereas RNA viruses infecting hyperthermophilic bacteria were classified into a new phylum *Artimaviricota* (realm *Riboviria*). In recognition of his extensive and valuable contributions to virus taxonomic developments in Study Groups and over the period of his EC membership, Stuart Siddell was honoured as a new life member of the ICTV. The ICTV has created a new strategy for disseminating information on taxonomy advances through annual open-access publication of citeable taxonomy proposal summaries from each ICTV Subcommittee. A collective total of 354 co-authors of the seven summaries were drawn from members of each Subcommittee, the EC, and a very large number of contributors from the wider virology community.

## Introduction

The International Committee on Taxonomy of Viruses (ICTV) follows an annual cycle of taxonomy updating, in which changes and additions to virus taxonomy proposed by the virology community are considered and implemented through the concerted effort of ICTV Study Groups, Subcommittees, and the Executive Committee. The ICTV classification of viruses provides a framework for the taxonomic placement of viruses at ranks from species to realm and furthermore regulates their taxon names and typography. The ICTV Statutes **(**https://ictv.global/about/statutes) describe the process through which taxonomic proposals are submitted to the ICTV Executive Committee (EC) and undergo review with input from ICTV Study Groups and Subcommittees, other interested virologists, and the EC. After final approval by the EC, proposals are placed on the ICTV website (https://ictv.global) for evaluation by the full ICTV membership and ratification by online voting.

### Proposal discussion and ratification

The annual EC meeting of the ICTV was held in Bari, Italy, from July 31 to August 2, 2024. The EC reviewed a total of 112 taxonomy proposals from six of the seven Subcommittees and three general proposals (no proposals were received from the Animal ssRNA + Subcommittee). Proposals were submitted by a total of 354 co-authors representing a substantial engagement in taxonomy by the wider virology community outside of the ICTV.

For assessment, 50 proposals had been “streamlined” through review by at least two EC members, including the SC Chair, prior to the meeting, and the remaining 65 proposals were discussed and voted upon by the EC. At the meeting, three proposals were rejected, and 13 proposals were accepted conditional on substantial revision and a second vote by the EC in November, 2024. Along with updated proposals subject to minor revision, 112 taxonomy proposals were placed on the ICTV website (https://ictv.global) for viewing by the full ICTV membership and the general public.

All proposals were voted on by the 169 members of the ICTV from January 27 to February 28, 2025. Voting was performed using an Excel-based form rather than online survey with the aim of providing more information about the proposal, greater flexibility in responses (including “Abstain”) and to discourage “Vote-for-All” responses often encountered in previous ratification votes. The voting form was accompanied by a summary of the taxonomy proposal in a single document (providing the title, authors, abstract, and tabulated taxonomy changes for each proposal) to facilitate review of the proposals when voting. A total of 106 out of 169 ICTV members (63%) voted on the proposals. Excluding seven erroneous votes against proposal 2024.001M.Alpharhabdovirinae_1ng_11nsp caused by a technical error on the form, proposals received a mean of 94.5 (range: 87–103) votes for acceptance, 11.5 (range: 3–19) abstentions, and 0.02 (only two votes) against. There was therefore an 89% vote for approval (range: 82–97%) or 100% (range: 99–100%) when abstentions were excluded. Following the vote of the membership, minor technical errors were identified in nine of the approved proposals. Using policies established by the EC, these errors were corrected, and the corrected proposals were approved and incorporated into Master Species List (MSL) 40 as version v2.

A summary of the taxonomy changes enacted by the proposals is provided in Table 1. Each proposal is cited and listed in the References [1–112] to acknowledge the authors’ efforts and to provide links to the specific proposal on the ICTV website. These documents and those from previous years are permanently available to provide full access to the text and listing of taxonomy changes made in each proposal (https://ictv.global/files/proposals/approved). All ratified proposals are furthermore published in a series of citeable summaries co-authored by all 354 contributors to the proposals [118, 122, 125 − 27, 129, 132]. A description of their format and how to cite taxonomy changes in publications is provided in an accompanying review article co-authored by the EC [113].

**Table 1:** Summary of ratified taxonomic changes in 2025

Rank	MSL39 Total^a^	New	Abolished	Moved	Renamed	Other	MSL40 v2 Total^b^	Net change
Realm	6	1	0	0	0	0	7	+ 1
Subrealm	0	0	0	0	0	0	0	0
Kingdom	10	1	0	1	0	0	11	+ 1
Subkingdom	0	0	0	0	0	0	0	0
Phylum	18	4	0	0	0	0	22	+ 4
Subphylum	2	2	0	0	0	0	4	+ 2
Class	41	8	0	4	1	0	49	+ 8
Subclass	0	0	0	0	0	0	0	0
Order	81	11	0	5	2	1	93	+ 12
Suborder	11	1	0	0	0	0	12	+ 1
Family	314	55	0	10	2	−1	368	+ 54
Subfamily	200	15	−2	15	1	0	213	+ 13
Genus	3,522	243	−2	100	4	6	3,768	+ 246
Subgenus	84	2	0	0	0	0	86	+ 2
Species	14,690	1,563	−38	105	273	0	16,213	+ 1,523

^a^Total number of taxa in the ICTV Master Species List (MSL) #39 prior to the 2025 ratification vote

^b^Total number of taxa after the 2025 ratification vote (listed in the ICTV MSL #40).

### Principal changes to virus taxonomy

The greatest number of new assignments were made at the species rank, with a current total of 16,213 species, almost all of which possess binomial names. This represents an increase of 1,523 over the previous year, and continues steadily rising totals of 10,434 (2022), 11,273 (2023), and 14,690 (2024). There were substantial increases in the numbers of genera and families (+ 246 and + 54, respectively), with new taxa being primarily assigned to bacterial viruses; additionally, several new families were established for archaeal and fungal viruses.

Eight new classes were established, including *Orpoviricetes* for bi-segmented fungal RNA viruses with non-canonical RNA-directed RNA polymerase (RdRP) motifs [68]. This assignment is provisional pending a potential future reclassification to a new phylum or even higher rank given the structural distinctiveness of the group’s RdRP hallmark gene. Several new classes were created as part of the re-organisation of the realm *Varidnaviria* (proposal 2024.010D.Varidnaviria_reorg; [58]) and the assignments of hakuzovirids, pleolipovirids, and anellovirids to new phyla ([4, 43, 59]; described below).

Higher rank changes included the addition of two subphyla, four phyla, one kingdom, and one realm, representing a substantial expansion in the diversity of classified viruses. A major change was the splitting of the established realm *Varidnaviria* to create a new realm, *Singelaviria*, now including the kingdom *Helvetiavirae* [58]. This was based on the recent recognition of independent evolutionary origins from cellular counterparts of double jelly-roll and single vertical jelly-roll major capsid proteins that are characteristic of double-stranded DNA viruses assigned to two kingdoms, *Bamfordvirae* and *Helvetiavirae*, respectively [114]. Furthermore, comparative analysis of the replication modules encoded by viruses in the realm *Varidnaviria* led to a major re-organization of this realm [114, 115], with five virus orders originally assigned to the kingdom *Bamfordvirae* being moved to a new kingdom, *Abadenavirae*. Finally, two new classes were established within phylum *Preplasmiviricota* to accommodate members of the reassigned family *Adenoviridae* [116] and the previously unclassified, environmentally ubiquitous “polinton-like” viruses [117, 118], and one new class was established within the phylum *Nucleocytoviricota* to accommodate small relatives of giant viruses [119, 120].

Proposal 2024.012D.Shotokuvirae_newphylum [59] addressed the lack of higher-rank assignments for anellovirids, small, single-stranded DNA viruses infecting a wide range of vertebrates. Although anellovirids lack the *Rep* gene typical of circovirids and other members of the phylum *Cressdnaviricota*, it was found that they encode a single jelly-roll capsid protein typical of single-stranded DNA viruses of the kingdom *Shotokuvirae* (realm *Monodnaviria*) [121, 122]. Accordingly, the newly created higher-rank taxonomic assignments for anellovirids include the order *Sanitavirales*, class *Cardeaviricetes*, and phylum *Commensaviricota*.

Archaeal viruses producing enveloped pleomorphic virions and infecting hyperthermophile prokaryotes of the class *Archaeoglobi* are highly distinct from, yet evolutionarily related to, pleolipovirids in the kingdom *Trapavirae* (realm *Monodnaviria*) [123]. In proposal 2024.004A.Thalassapleoviridae_newphylum [4, 121], Archaeoglobus veneficus pleomorphic virus 1 (AvPV1)-like viruses have been assigned to an entirely separate lineage comprising a new phylum, *Calorviricota*, in the kingdom *Trapavirae*, class *Caminiviricetes*, order *Ageovirales*, and family *Thalassapleoviridae*, with three genera and five species.

A new phylum, *Artimaviricota*, and included lower ranks (*Furtirnaviricetes*, *Divaquavirales, Hakuzoviridae, Atsuirnavirus*), was established in the kingdom *Orthornavirae* (realm *Riboviria*) for the hyperthermophilic, bi-segmented bacterial hot spring RNA virus 1 [43]. This assignment was based on its highly divergent RdRP sequence and deduced structure that groups phylogenetically apart from homologues in the other six currently assigned orthornavirian phyla [124].

Two new orders of bacterial viruses were established. The family *Autographiviridae* was elevated to the order *Autographivirales* and includes four newly created families [49]. The order encompasses bacterial viruses with podovirus morphology that encode a large single-subunit DNA-directed RNA polymerase. The Lak megaphages, which have been shown to be prevalent across a diversity of gut microbiomes through genome-resolved metagenomics, were assigned to a new order, *Grandevirales* [20]. These viruses possess some of largest known caudoviricete genomes and are characterised by an alternative genetic code in which the TAG stop codon is repurposed to encode glutamine.

Among the three proposals in the General category, Professor Stuart Siddell was nominated as a Life Member of the ICTV [71], based on his long service as a member of ICTV Study Groups, Subcommittees, and the Executive Committee and his key role in helping to develop and modernize guiding principles used to create virus taxonomy.

### Implementation and access

The latest set of proposals approved by the EC was made available on the ICTV website in April, 2025 at https://ictv.global/files/proposals/approved (all proposals combined into a single zip file) and also in a directory at https://ictv.global/files/proposals/approved where links provide access to proposals indexed by virus group and Subcommittee.

Updated versions of the Master Species List (release 40 version v2), which lists all currently approved taxa (Table 1), can be accessed on the ICTV website at https://ictv.global/msl. A similarly updated release 40 (March 27, 2025) of the Virus Metadata Resource (VMR) is located at https://ictv.global/vmr. This provides details of exemplar virus isolates for each species, including GenBank accession numbers.

Summaries of the ratified proposals (described in reference [113, 120]) from six of the seven ICTV Subcommittees and for general proposals are available [125–131].

## Data Availability

All Taxonomy Proposals and all other ICTV resources mentioned in this article are freely available at the ICTV website (https:/ictv.global/)
